# Semi-elemental versus polymeric formula for enteral nutrition in critically ill patients: a secondary analysis of a multicenter cluster-randomized controlled trial

**DOI:** 10.3389/fnut.2025.1587270

**Published:** 2025-07-23

**Authors:** Wei Wei, Wen Lu, Guofeng Chen, Jindan Gao, Jun Zhang, Defeng Zhang, Ruiqin He, Jingjing Huang, Rong Cai, Rongrong Yuan, Xun Wang, Jinxia Yu, Zilong Li, Lu Ke, Lin Gao, Zhengquan Wang

**Affiliations:** ^1^Department of Emergency Medicine, Yangming Hospital Affiliated to Ningbo University (The People’s Hospital of Yuyao City), Yuyao, Zhejiang, China; ^2^Department of Critical Care Medicine, Jinling Hospital, Medical School of Nanjing University, Nanjing, China

**Keywords:** critically ill, enteral nutrition, semi-elemental, gastric feeding, gastrointestinal intolerance

## Abstract

**Objectives:**

Semi-elemental enteral nutrition (EN) might theoretically improve gastrointestinal tolerance in critically ill patients; however, it is associated with an increased risk of diarrhea when delivered postpylorically. This study aimed to assess whether the use of semi-elemental formula compared to polymeric formula may provide benefits in patients receiving gastric tube feeding.

**Methods:**

This is a *post-hoc* analysis of data from a multicenter, cluster-randomized, controlled, investigator-initiated trial (NEED trial). Patients were eligible if they stayed in the participating intensive care units (ICUs) and received gastric EN exclusively during the first week of enrollment. A directed acyclic graph (DAG) was used to identify potential confounders. Propensity score matching (PSM) was applied to control for the detected confounders. The primary outcome was the incidence of intolerance-related symptoms, including nausea/vomiting, aspiration, abdominal distension/pain, and diarrhea.

**Results:**

PSM created 516 matched pairs from 1,548 eligible patients. The incidence of abdominal distension/pain was significantly lower in the semi-elemental group compared to the polymeric group (9.1% versus 13.8%, risk ratio, 0.66; 95% CI, 0.46 to 0.93; *p* = 0.027). No significant differences were observed in the incidence of nausea/vomiting, aspiration, or diarrhea between groups.

**Conclusion:**

In critically ill patients receiving EN via gastric access, the semi-elemental formula was associated with a reduced incidence of abdominal distension/pain, but not with an increased incidence of diarrhea, compared to the polymeric formula.

**Clinical trial registration:**

https://www.isrctn.com/ISRCTN12233792?q=ISRCTN12233792&filters=&sort=&offset=1&totalResults=1&page=1&pageSize=10, Identifier ISRCTN12233792.

## Introduction

Critically ill patients admitted to intensive care units (ICUs) usually experience a marked metabolic disorder and increased protein catabolism (1, 2), resulting in a high risk of energy and protein deficits associated with higher mortality, more infectious morbidity, and other unfavorable outcomes (3, 4). Nutrition therapy, especially sufficient enteral nutrition (EN), is the cornerstone to correct these deficits (5). However, gastrointestinal intolerance during EN is common in critically ill patients and could lead to delay or interruption of EN (6–8). EN intolerance is clinically recognized by the presence of elevated gastric residuals, nausea, vomiting, abdominal distension, and diarrhea and is considered to be associated with aspiration and even inhalation pneumonia (6, 7, 9).

For the majority of ICU patients requiring EN, it is recommended to use a standard polymeric whole protein formula as a first-line treatment (10), considering its cost-effectiveness compared to a semi-elemental formula (11). However, semi-elemental formulas contain small peptides and predominantly medium-chain triglycerides (MCTs), and the use of semi-elemental over polymeric formula (containing intact protein, complex carbohydrates, and long-chain triglycerides) presents several theoretical advantages (12). It is believed that semi-elemental diets are more easily assimilated, absorbed, and better tolerated. It has been shown that the use of semi-elemental solutions was associated with reduced regurgitation, improved gastric emptying, and improved EN tolerance (12–14).

However, semi-elemental formulas have been known to increase the risk of diarrhea, which may be attributed to the higher osmolar load incurred by semi-elemental contents (15). The greater the extent of protein hydrolysis, the higher the osmolality and the greater the risk of causing osmotic diarrhea (16). Physiologically, a hyperosmolar formula can trigger the gut to secrete additional fluid to dilute it to a tolerable osmolality, potentially causing diarrhea, particularly when infused postpylorically (e.g., via duodenal or jejunal access) (17). For patients on gastric feeding, which is the case in most critically ill patients (18), this may not be a concern since the solution would not directly stimulate the gut. Furthermore, the hyperosmolar solution can mix with gastric contents before being released to the gut, thereby reducing osmolality (19). However, there are few studies in the literature comparing different formulas in exclusive gastric feeding patients. This study aimed to assess whether the use of semi-elemental formula compared to polymeric formula was associated with better feeding tolerance in critically ill patients receiving EN via gastric access.

## Methods

### Study design and patients

This study is a secondary analysis of a multicenter, cluster-randomized controlled trial (RCT) (NEED trial) (ISRCTN Registry: ISRCTN12233792) that aimed to assess the impact of an evidence-based feeding guideline on clinical outcomes in critically ill patients. The study design, methodology, population, and main results of the NEED trial have been reported previously (20). A total of 2,772 patients were recruited from 90 ICUs across China between March 2018 and July 2019. The participating ICUs were randomized with a 1:1 ratio to implement the feeding guideline (guideline group) or to remain unaware of the guideline content (control group). The trial found no difference in the primary outcome of 28-day mortality between the guidelines and the control groups. Data storage and academic use of de-identified data after the trial were covered in ethical approval, and informed consent was obtained from the patients or their surrogates prior to enrollment.

This secondary analysis is performed in a subgroup of the NEED trial participants. Patients were eligible if they stayed in participating ICUs and received gastric EN exclusively during the first week of enrollment. Patients who did not have EN initiated within the first 4 days, mainly received oral diets, had missing data to identify the type of EN formula received, or received mixed use of semi-elemental and polymeric formulas during the first week, were excluded. The semi-elemental formula used in this study was primarily Peptisorb® (Nutricia, Netherlands), while other products were classified as polymeric (whole-protein) formulas.

### Data collection and outcomes

All data were extracted from the trial electronic database, including de-identified patient characteristics, daily nutritional therapy, and main clinical outcomes. The baseline characteristics include age, sex, body mass index (BMI), Sequential Organ Failure Assessment (SOFA) score, Acute Physiology and Chronic Health Evaluation II (APACHE II) score, modified Nutrition Risk in the Critically Ill (mNUTRIC) score, acute gastrointestinal injury (AGI) score, source of ICU admission, primary admission diagnosis, number of comorbidities, and the requirement of organ support at enrollment. Daily nutritional variables included the EN formula received (semi-elemental or polymeric), daily energy intake, and daily protein intake. Nutritional intake was recorded for 7 consecutive days after enrollment. The total amount of energy intake was calculated from EN, parenteral nutrition (PN), and dextrose-containing intravenous fluids. The total amount of protein intake was calculated from EN, PN, and intravenous amino acid solutions.

The primary outcome was the incidence of intolerance-related symptoms from day 5 to day 7 after enrollment, including nausea/vomiting (gastric contents located outside the mouth), aspiration (gastric contents detected in the airway), abdominal distension/pain, and diarrhea, as defined by a previously published self-developed feeding intolerance score (20). Diarrhea was defined as more than 3 unusually loose or watery stools (total amount ≥250 mL) per day. Secondary outcomes included 28-day mortality and ICU-free days within 28 days, which were defined as the number of days alive and out of the ICUs.

### Statistical analyses

The Shapiro–Wilk test was used to test the normality of continuous variables. Continuous normally distributed data were reported as mean ± standard deviation (SD). Skewed continuous data were reported as median (interquartile range, IQR). Categorical data were summarized by frequencies and percentages. The differences between the two groups were compared by the Student’s t-test (normally distributed) or Wilcoxon rank-sum test (skewed data) for continuous variables and the χ^2^ test or Fisher’s exact test for categorical data.

**Figure 1:**
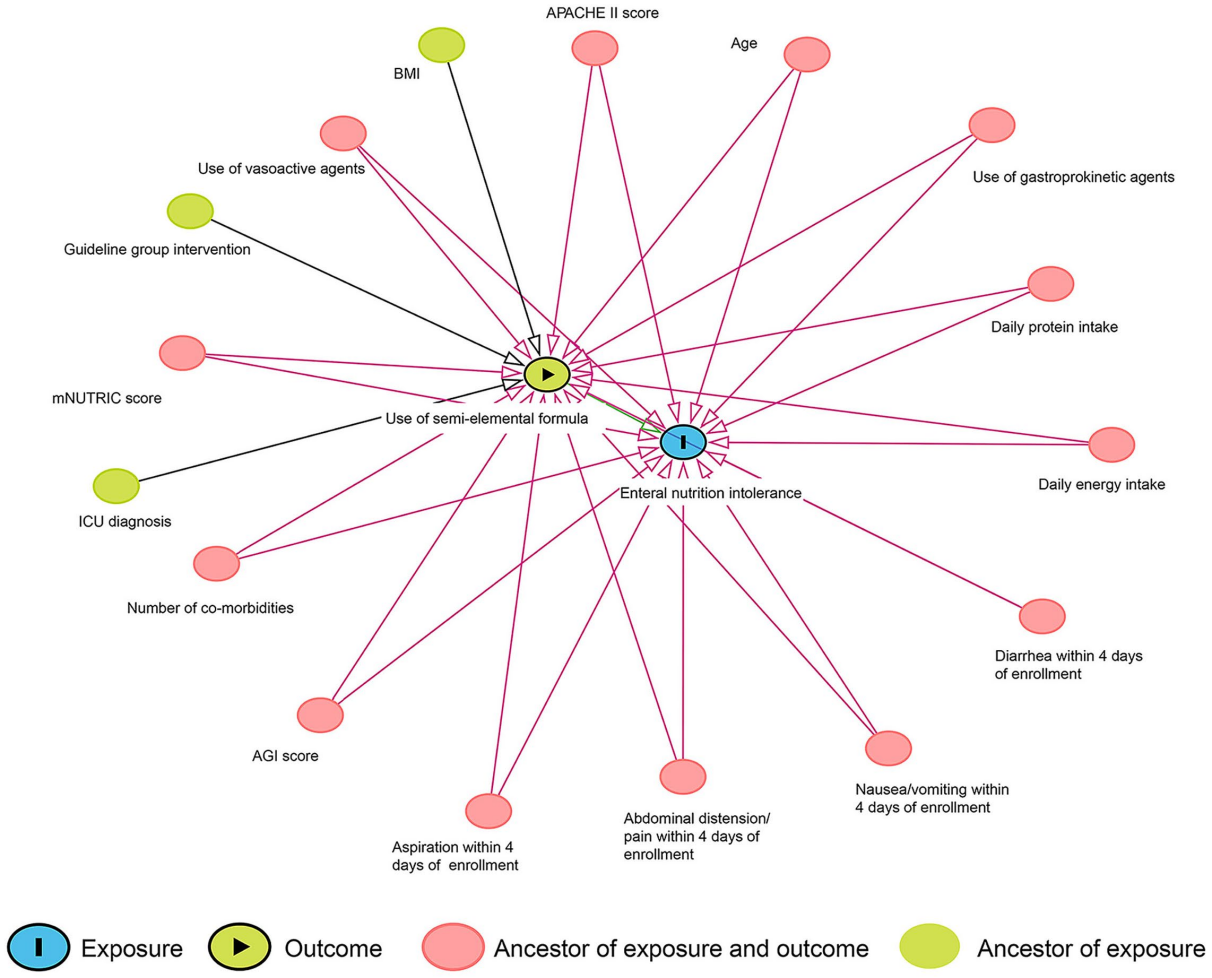
Directed acyclic graph of the associations between the use of the semi-elemental formula and enteral nutrition intolerance.

A directed acyclic graph (DAG) was used to identify the minimum adjustment required for the confounder control (drawn in DAGitty 3.0, Figure 1). The current literature was used as the basis for elaborating the interrelations between the semi-elemental formula and EN intolerance. As a result, age, BMI, ICU diagnosis, study intervention (guideline group), APACHE II score, AGI score, mNUTRIC score, number of comorbidities, use of vasoactive agents, use of gastroprokinetic agents, EN intolerance within 4 days of enrollment, daily energy intake, and daily protein intake were identified as confounders. Early intolerance symptoms (days 1–4), which likely reflect the severity of critical illness rather than the effects of the formula, were treated as potential confounders for later-stage intolerance (days 5–7). Propensity score matching (PSM) analysis was used to control the above-detected confounders. Patients who received the semi-elemental formula were matched at a 1:1 ratio with patients who received the polymeric formula using their propensity score. The multicollinearity between the potential confounding variables was checked by the variance inflation factor. One-to-one nearest neighbor matching with a caliper width of 0.20 was used in the PSM. The standardized mean difference was used to assess the balance of baseline covariates between treatment groups in both the matched and unmatched samples. A standardized mean difference greater than 0.1 and a two-sided *p-*value of less than 0.05 indicated a significant imbalance in the baseline covariates.

For the matched pairs, the differences in binomial outcomes between groups were assessed with risk differences and 95% confidence intervals (CIs). The differences in continuous outcomes were evaluated with median differences, and 95% CIs were also calculated. The *p*-value was tested using the Wilcoxon signed-rank test (continuous outcomes) and the McNemar test (binomial outcomes) for matched data.

All analyses were performed using a two-sided test, with a significance level of *p* = 0.05, and presented with two-sided 95% CIs. Analyses were performed using R software, version 4.4.1 (R Project for Statistical Computing).

## Results

### Patient characteristics

**Figure 2:**
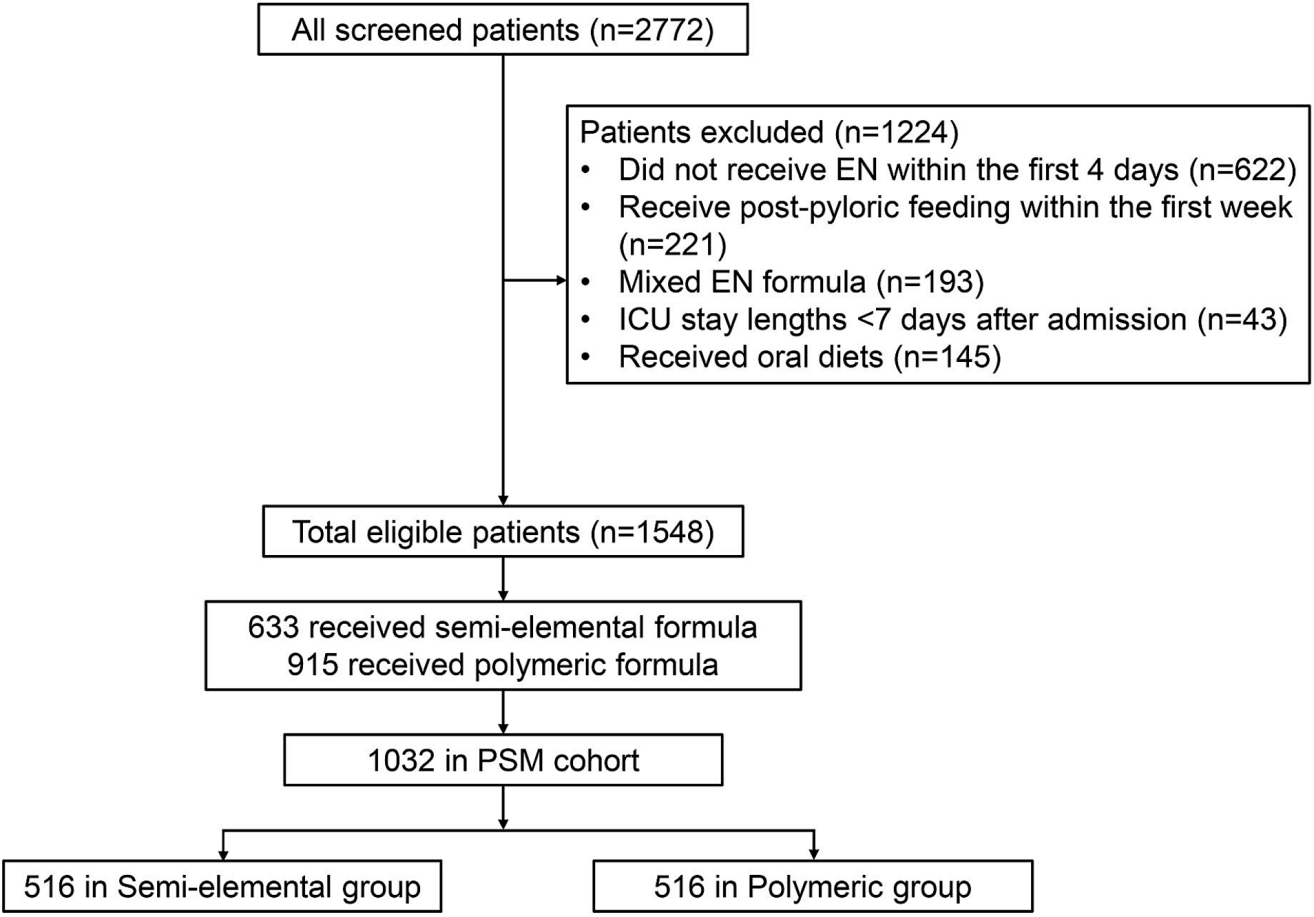
Study flow chart.

A total of 1,548 eligible patients were included in the analysis (Figure 2). Within this cohort, 633 patients (40.9%) received semi-elemental formula and 915 patients (59.1%) received polymeric formula within the first 7 days of enrollment. Baseline characteristics, clinical features, and nutrition therapy are summarized in Table 1. Approximately half of the study patients were admitted to the ICUs for respiratory reasons (43.8%), followed by cardiovascular (26.0%) and neurological (17.7%) conditions. The majority of study patients (72.9%) were on mechanical ventilation at enrollment. Patients in the semi-elemental formula group had a significantly higher AGI score, and 23.9% of patients were classified as AGI II or higher compared to 14.4% in the polymeric formula group (*p* < 0.001).

**Table 1:** Baseline characteristics and clinical features of included patients.

Variables	Total (*n* = 1,548)	Participants before matching, No. (%)		SMD	Participants after matching, No. (%)		SMD
		Semi-elemental group (*n* = 633)	Polymeric group (*n* = 915)		Semi-elemental group (*n* = 516)	Polymeric group (*n* = 516)	
Age, year, median (IQR)	63 (50–75)	62 (49–73)	65 (51–76)	0.174	63 (50–74)	63 (48–75)	0.037
Male, *n* (%)	1,029 (66.5)	425 (67.1)	604 (66.0)	0.024	342 (66.3)	352 (68.2)	0.041
BMI, kg/m^2^, median (IQR)	22.5 (20.8–24.5)	22.5 (20.8–24.1)	22.9 (20.8–24.8)	0.148	22.5 (20.8–24.2)	22.5 (20.8–24.6)	0.038
APACHE II score, median (IQR)	19 (14–23)	19 (14–23)	19 (14–23)	0.027	19 (14–23)	18 (14–23)	0.001
SOFA score, median (IQR)	7 (5–10)	7 (6–10)	7 (5–10)	0.161	7 (5–10)	7 (5–10)	0.014
mNUTRIC score, median (IQR)	5 (3–6)	5 (3–6)	5 (3–6)	0.019	5 (3–6)	4.5 (3–6)	<0.001
Number of co-morbidities median (IQR)	2 (1–3)	2 (1–3)	2 (1–3)	0.004	2 (1–3)	2 (1–3)	0.042
Study interventions, *n* (%)				0.263			0.047
Guideline group	850 (54.9)	396 (62.6)	454 (49.6)		305 (59.1)	293 (56.8)	
Control group	698 (45.1)	237 (37.4)	461 (50.4)		211 (40.9)	223 (43.2)	
AGI score, *n* (%)				0.272			0.051
AGI I	1,265 (81.7)	482 (76.1)	783 (85.6)		427 (82.8)	417 (80.8)	
AGI II	233 (15.1)	121 (19.1)	112 (12.2)		76 (14.7)	84 (16.3)	
AGI III	36 (2.3)	18 (2.8)	18 (2.0)		13 (2.5)	15 (2.9)	
AGI IV	14 (0.9)	12 (1.9)	2 (0.2)		0	0	
Primary admission diagnosis, *n* (%)				0.201			0.040
Respiratory	678 (43.8)	263 (41.5)	415 (45.4)		239 (46.3)	232 (45.0)	
Cardiocirculatory	403 (26.0)	196 (31.0)	207 (22.6)		135 (26.2)	144 (27.9)	
Neurologic	274 (17.7)	96 (15.2)	178 (19.5)		80 (15.5)	79 (15.3)	
Other	193 (12.5)	78 (12.3)	115 (12.6)		62 (12.0)	61 (11.8)	
Requirement of organ support, *n* (%)							
MV	1,129 (72.9)	486 (76.8)	643 (70.3)	0.148	393 (76.2)	373 (72.3)	0.089
CRRT	160 (10.3)	79 (12.5)	81 (8.9)	0.118	62 (12.0)	51 (9.9)	0.068
Vasoactive agents	471 (30.4)	229 (36.2)	242 (26.4)	0.211	162 (31.4)	169 (32.8)	0.029
Feeding intolerance within 4 days of enrollment, *n* (%)							
Abdominal distension/pain	377 (24.4)	167 (26.4)	210 (23.0)	0.080	132 (25.6)	129 (25.0)	0.013
Nausea/vomiting	99 (6.4)	51 (8.1)	48 (5.2)	0.113	33 (6.4)	38 (7.4)	0.038
Aspiration	16 (1.0)	7 (1.1)	9 (1.0)	0.012	6 (1.2)	7 (1.4)	0.017
Diarrhea	184 (11.9)	86 (13.6)	98 (10.7)	0.088	69 (13.4)	66 (12.8)	0.017
Use of gastroprokinetic agents, *n* (%)	95 (6.1)	37 (5.8)	58 (6.3)	0.021	29 (5.6)	33 (6.4)	0.033
Daily energy intake volume during the first 4 days, kcal/kg/d, median (IQR)	12.7 (8.0–18.1)	11.1 (6.5–16.4)	14.1 (9.4–19.1)	0.389	12.0 (7.3–17.2)	12.4 (8.0–16.9)	0.001
Daily protein intake volume during the first 4 days, g/kg/d, median (IQR)	0.47 (0.29–0.67)	0.42 (0.24–0.62)	0.50 (0.33–0.69)	0.264	0.46 (0.28–0.67)	0.45 (0.29–0.65)	0.010

IQR, interquartile range; SMD, standardized mean difference; BMI, body mass index; APACHE II, Acute Physiology and Chronic Health Evaluation II; SOFA, Sequential Organ Failure Assessment; mNUTRIC, modified Nutrition Risk in the Critically ill; AGI, acute gastrointestinal injury; CRRT, continuous renal replacement therapy; MV, mechanical ventilation.

**Figure 3:**
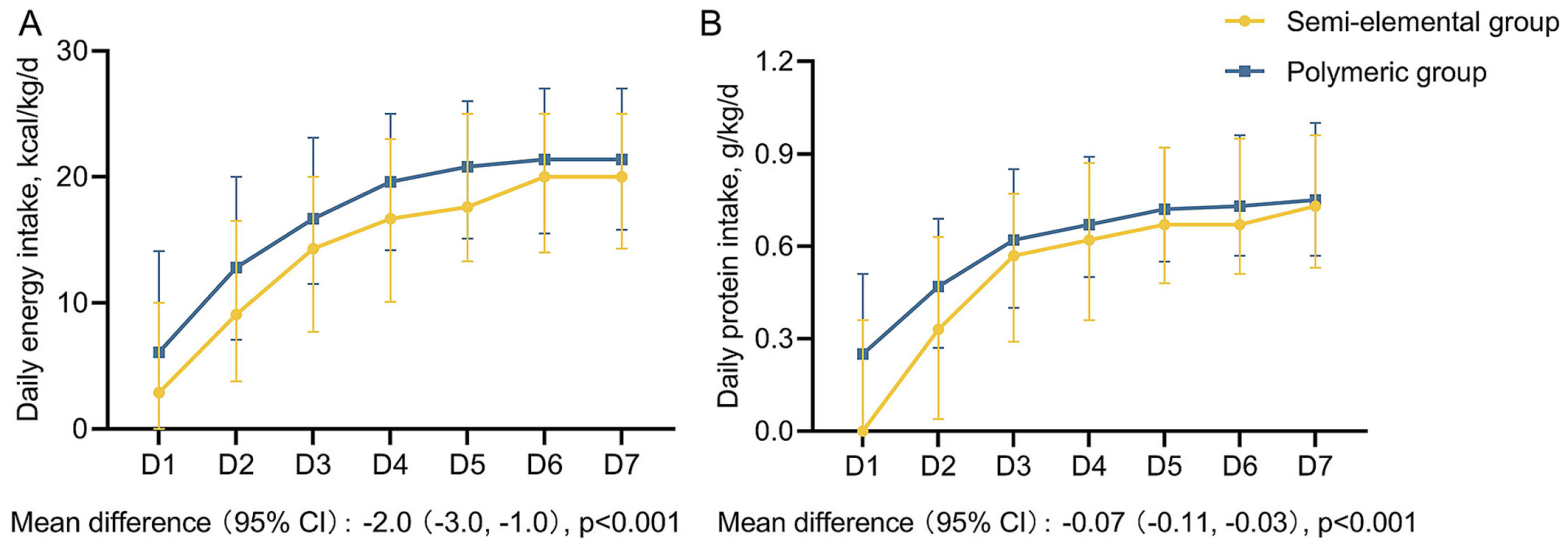
Daily energy and protein intake during the first week among two groups. **(A)** Daily energy intake, **(B)** Daily protein intake. The boxes represent medians, and the error bar represents the interquartile range.

The median daily energy and protein intakes for the first week in the two groups are shown in Figure 3. Both the median daily energy and protein intakes during the first week were significantly higher in the polymeric formula group than in the semi-elemental group. The semi-elemental group had significantly lower median daily energy and protein intakes compared to the polymeric group: 15.0 versus 17.7 kcal/kg/day (mean difference [MD] = −2.0; 95% CI, −3.0 to −1.0; *p* < 0.001) and 0.57 versus 0.64 g/kg/day (MD = −0.07; 95% CI, −0.11 to −0.03; *p* < 0.001), respectively.

### Propensity score matching

One-to-one PSM created 516 matched pairs, and the imbalance in the covariates between the semi-elemental and polymeric groups was significantly diminished after PSM. The distributions of the propensity scores and the balance of the covariates before and after matching are shown in Supplementary Figure 1.

### Primary and secondary outcomes

**Figure 4:**
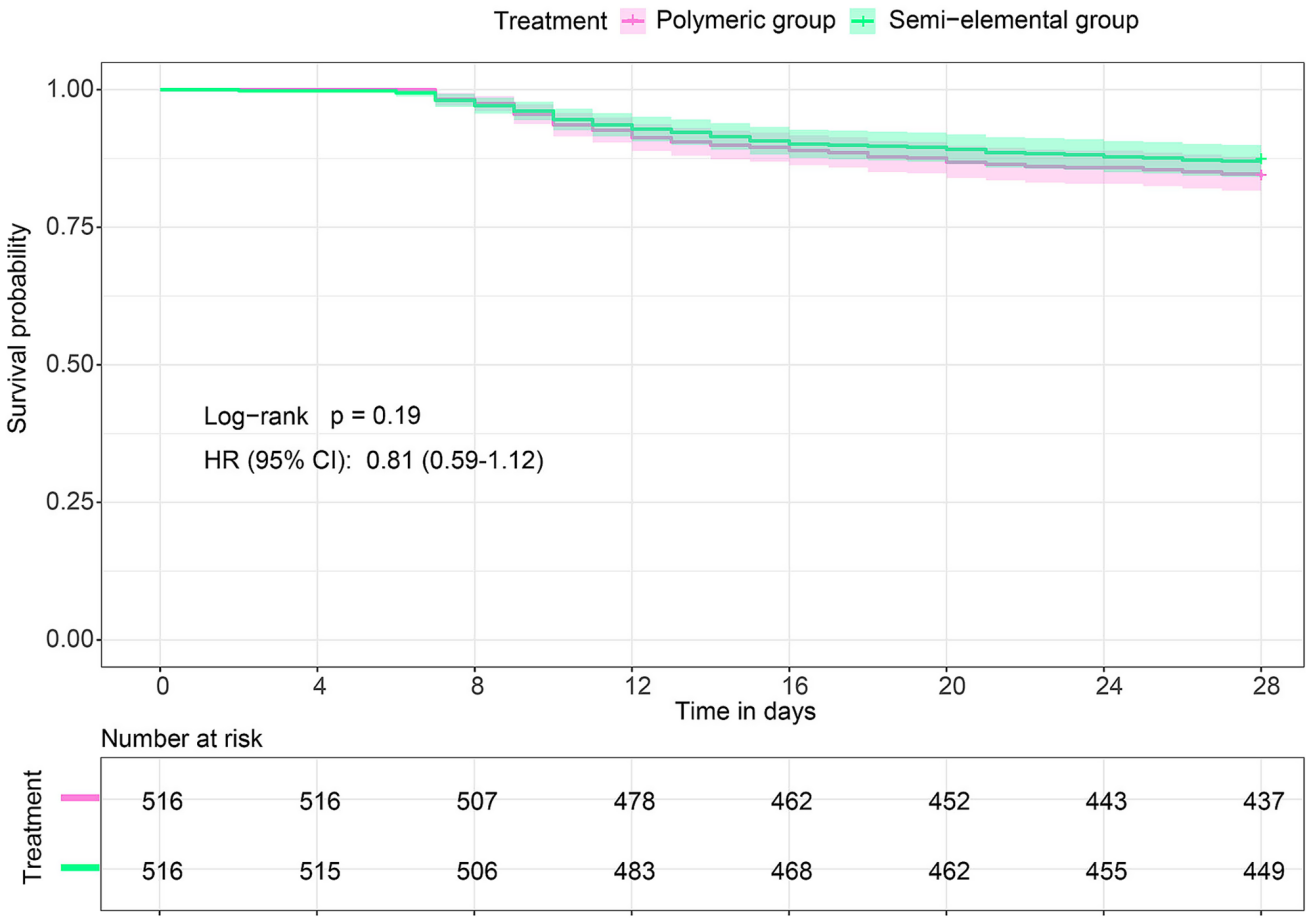
Survival curves. HR denotes hazard ratio; CI denotes confidence interval.

Data on feeding intolerance and other clinical outcomes are shown in Table 2. In the matched cohort, the incidence of abdominal distension/pain was significantly lower in the semi-elemental group compared to the polymeric group (9.1% versus 13.8%, risk ratio, 0.66; 95% CI, 0.46 to 0.93; *p* = 0.027). No significant difference was observed in the incidence of nausea/vomiting (4.7% versus 4.7%, risk ratio, 1.0; 95% CI, 0.57 to 1.74; *p* = 1.0), aspiration (2.7% versus 1.4%, risk ratio, 2.0; 95% CI, 0.84 to 5.24; *p* = 0.189), or diarrhea (15.7% versus 14.3%, risk ratio, 1.09; 95% CI, 0.82 to 1.47; *p* = 0.616) between the two groups. For secondary outcomes, there were no differences in ICU-free days within 28 days or in 28-day mortality between the groups. The Kaplan–Meier curves and Cox proportional hazards models also showed no difference in the survival rate of patients between the two groups (hazard ratio [HR], 0.81; 95% CI, 0.59 to 1.12; log-rank *p* = 0.19) (Figure 4).

**Table 2:** Primary and secondary outcomes.

Outcomes	Semi-elemental group (*n* = 516)	Polymeric group (*n* = 516)	Median difference / Risk ratio^§^ (95% CI)	*p*-value
Primary outcomes				
Abdominal distension/pain, *n* (%)	47 (9.1)	71 (13.8)	0.66 (0.46, 0.93)	0.027
Nausea/vomiting, *n* (%)	24 (4.7)	24 (4.7)	1.00 (0.57, 1.74)	1.0
Diarrhea, *n* (%)	81 (15.7)	74 (14.3)	1.09 (0.82, 1.47)	0.616
Pulmonary aspiration, *n* (%)	14 (2.7)	7 (1.4)	2.00 (0.84, 5.24)	0.189
Secondary outcomes				
ICU-free days within 28 days, median (IQR)	16 (10, 19)	17 (11, 20)	−0.5 (−1.5, 0.5)	0.215
28-day mortality, *n* (%)	67 (13.0)	82 (15.9)	0.82 (0.60, 1.10)	0.210

CI, confidence interval; ICU, intensive care unit.

§ Median difference was shown for continuous variables, and risk ratio was shown for categorical variables.

## Discussion

In the present study, patients fed with the semi-elemental formula had higher initial acute gastrointestinal injury scores at enrollment and received significantly lower daily energy and protein intake during the first week than those fed with polymeric formula. After propensity score matching, the use of a semi-elemental formula—compared to the polymeric formula—was associated with a reduced incidence of abdominal distension/pain, but not with an increased incidence of diarrhea in critically ill patients receiving gastric feeding. However, this improvement in gastrointestinal tolerance was not associated with a mortality benefit.

Previous clinical trials have shown that semi-elemental formulas do not appear to be superior regarding gastrointestinal tolerance and other patient-centered outcomes when prescribed to unselected ICU patients (21, 22). Given the lack of a demonstrated clinical benefit and the higher cost compared to polymeric formula, both the American Society for Parenteral and Enteral Nutrition (ASPEN) and the European Society for Clinical Nutrition and Metabolism (ESPEN) guidelines recommend against the use of the semi-elemental formula as a first-line EN prescription in ICU patients (10, 23). However, the nutritional and clinical benefits of semi-elemental formulas have been shown in patients across several specific care settings. For instance, in both ICU patients with acute gastrointestinal (GI) injury (24) and those receiving EN after abdominal surgery (25), the use of peptide-based EN formulas was associated with a lower incidence of gastric retention compared to the standard formula. In addition, a meta-analysis of small peptide formulas versus standard polymeric formulas in critically ill patients with acute GI injury showed that, although there was no improvement in feeding intolerance, the use of small peptide formulas was associated with higher serum albumin levels and shorter ICU and hospital stays (13). Our data showed that there was a higher percentage of GI dysfunction at enrolment in patients fed with the semi-elemental formula compared with the polymeric formula, suggesting that the use of the elemental formula is a common practice when clinicians encounter GI dysfunction.

It is assumed that most, if not all, patients with GI dysfunction have varying degrees of malabsorption and/or maldigestion; therefore, they may benefit from elemental or semi-elemental formulas (26). This assumption is based on the physiological observation that the dipeptides and tripeptides of semi-elemental formulas have specific uptake transport mechanisms and are believed to be absorbed more efficiently than whole proteins, which are the nitrogen sources in polymeric formulas (27). In addition to protein, the improvement in fat digestion and absorption was also believed to relieve GI intolerance (28). A multicenter RCT found that, compared to a standard enteral formula, an enteral diet rich in medium-chain triglycerides (MCTs), carnitine, and taurine could reduce the incidence of feeding intolerance, particularly abdominal distension (29). MCTs are the predominant fat source in the semi-elemental formula and can be absorbed directly across the small intestinal mucosa into the portal vein in the absence of lipase or bile salts (27). We hypothesize that it is the short peptides and MCTs in the semi-elemental formula that require minimal digestive function, potentially reducing the metabolic burden on the GI tract and subsequently reducing the risk of feeding intolerance. Despite the purported advantage of better absorption of semi-elemental formulas, it is worth noting that the actual absorption of the EN formulas cannot be accurately and routinely assessed in current clinical practice, and whether the improved absorption kinetics of these semi-elemental feeds could translate into clinical benefits remains to be further investigated.

Concerns about the osmolality of EN formulas as a relevant contributor to EN intolerance, particularly diarrhea, are common among clinical practitioners, although not justified by current evidence. A previous study investigated the effect of hypertonic gastric tube feeding on diarrhea in hospitalized patients and found that hypertonic (690 mOsm), low-residue, lactose-free tube feeding did not cause diarrhea in non-ICU patients but did cause diarrhea in a small statistically insignificant percentage of mechanically ventilated patients (3/24, 12.5%) (30). This finding suggests that the use of hyperosmolar EN via gastric access was not associated with increased diarrhea, even at osmolalities as high as 690 mOsm. Similarly, another study that compared the effects of small-peptide and whole-protein ENs on diarrhea (22) found that diarrhea in tube-fed patients is most often caused by factors such as liquid medications containing sorbitol or other offending ingredients rather than tube feeding itself, regardless of the formulas given.

This study had several limitations. First, although we controlled for available variables associated with the semi-elemental formula use or EN intolerance, there may be unmeasured influential variables that were not controlled for in our propensity score matching model. Second, given the *post-hoc* nature of the analysis, causality cannot be inferred, and the conclusions should be interpreted with caution. Third, we did not control for potential confounders of diarrhea, such as treatment with antibiotics, use of diarrhea-causing medications, or other offending agents, due to a lack of relevant data. Fourth, while energy and protein intake were used as indicators of nutritional adequacy, analysis of biochemical nutritional markers was limited by inconsistent data availability across centers and by possible inter-laboratory variability. Finally, since semi-elemental formulas are typically more expensive than standard polymeric formulas, a formal cost–benefit analysis was not feasible due to the absence of systematically collected economic data. Future studies should incorporate cost-effectiveness evaluations.

## Conclusion

In this *post-hoc* analysis of a multicenter RCT, we found that, among critically ill patients receiving EN via gastric access, the semi-elemental formula was associated with a reduced incidence of abdominal distension/pain, but not with an increased incidence of diarrhea, compared to the polymeric formula. Further RCTs are warranted to confirm our findings.

## Data Availability

The raw data supporting the conclusions of this article will be made available by the authors, without undue reservation.

## Supplementary material

SUPPLEMENTARY FIGURE 1 Distribution of propensity scores **(A)** and the balance of the covariates after propensity score matching **(B)**.
