# Supplementary figures and images for: Semi-elemental versus polymeric formula for enteral nutrition in critically ill patients: a secondary analysis of a multicenter cluster-randomized controlled trial
Source: Front Nutr. 2025 Jul 23;12:1587270. doi: 10.3389/fnut.2025.1587270 (PMC12325058; doi:10.3389/fnut.2025.1587270)

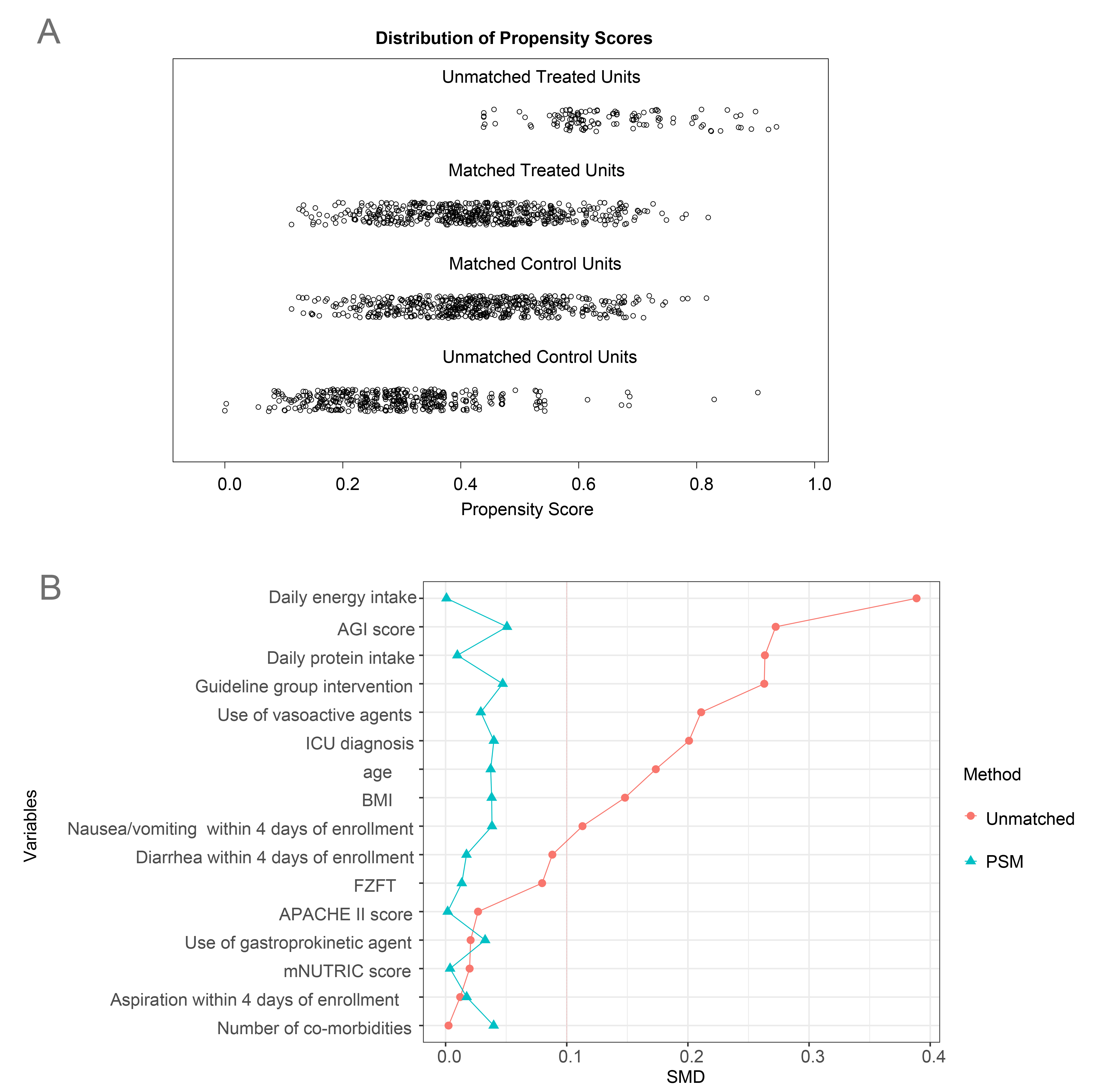

Supplement: SUPPLEMENTARY FIGURE 1 — Distribution of propensity scores (A) and the balance of the covariates after propensity score matching (B). [file Image_1.jpeg]
